# 

**Published:** 1899-03

**Authors:** 


### SELECTIONS.

Article I.—Our Relations with our American Confreres Living and Practicing abroad ........ 481
" II.—Nature's Methods of Defense. L. P. Bethel.......... 486
" III.—Artificial Dentures in their Relation to the Speaking or the Singing Voice. Dwight L. Hubbard, M. D... 491
" IV.—Anesthesia by Rapid Breathing. Dr. W. G. A. Bonwill........ 498
" V.—The Use of Formalin. Dr. H. Jerome Allen........... 500
" VI.—An Easy Method of Refining Gold. Dr. A. D. Hooker. 503
" VII.—The Importance and Manner of Sterilization of Dental Instruments. L. A. Meyer, D. D. S.............. 505
" VIII.—A New Dentinal Obtundent ........ 510
" IX—Degeneration and Decay of the Oral Tissues from Lack of Exercise. S. B. Dewey, D. D. S., M. D.... 512
" X.—Alloy As a Filling Material. W. H. Chilson, D. D. S. 518

### MONTHLY SUMMARY.

Instruments to Carry Outside........ 522

A New Way of Preserving Meat........ 523

Thoughtless Mothers ........ 524

Dental Detection of Criminals ........ 524

Filling vs Crown........ 526

Surgical Treatment of Abscess ........ 526

Roots and Bridges ........ 527

Treatment of Decalcified Dentine in Bottom of Cavity........ 527

In Taking ........ 528

Annealing Gold........ 528

To Remove Plaster from Vulcanite plates ........ 528

Soak it........ 528

